# Molecular mechanism of thyroid hormone action in carcinogenesis

**DOI:** 10.1186/1756-6614-6-S2-A48

**Published:** 2013-04-05

**Authors:** Agnieszka Piekiełko-Witkowska

**Affiliations:** 1 Department of Biochemistry and Molecular Biology, Centre of Postgraduate Medical Education, Warsaw, Poland

## 

Thyroid hormones, 3,5,3'5'-tetraiodothyronine (thyroxine) and 3,5,3'-triiodothyronine (T3) contribute to the regulation of key cellular processes, including proliferation, differentiation, apoptosis and metabolism. Thus, deregulation of thyroid hormone pathway can result in serious disturbances of cellular physiology such as those observed in tumoral transformation. The intra- and extracellular concentrations of thyroid hormones (TH) are regulated by three types of iodothyronine deiodinases (DIO1, DIO2, and DIO3). These enzymes catalyze two types of reactions: deiodination in position 5' of iodothyronines (catalyzed by DIO1 and DIO2), resulting in activation of thyroid hormones, and deiodination in position 5 of iodothyronines (catalyzed by DIO1 and DIO3), which results in inactivation of thyroid hormones. Changes in expression and activity of iodothyronine deiodinases result in modulations of thyroid hormone concentrations and thus influence cellular processes. The actions of thyroid hormone are mediated by thyroid hormone receptors (TRs) which influence intracellular processes via modulation of expression of target genes. In this process, known as genomic mechanism of TH action, thyroid hormone receptors act as transcription factors whose activity is modulated by the presence of ligand, T3. TH can also influence cellular processes via non-genomic pathways in which TH modulate activity of membrane or intracellular receptors and activate different intracellular signaling pathways, involving protein kinases, for instance PI3K, and Akt/PKB.

In the process of carcinogenesis the signaling pathway of thyroid hormones is disturbed. This is illustrated by observations of mutations and deregulated expression of thyroid hormone receptors, altered expression and activity of iodothyronine deiodinases as well as decreased intracellular concentrations of T3 in tumours. Whether these changes play a causative role in the process of carcinogenesis is currently a matter of discussion. In vitro studies in cancer cell lines and mouse models showed that increased expression of DIO3 together with enhanced proteasomal degradation of DIO2 in basal cell carcinoma stimulates tumoral proliferation. Moreover, thyroid hormone receptor beta-1 was shown to act as a powerful suppressor of tumour invasiveness and metastasis in mouse models of breast cancer and hepatocarcinoma. These studies suggest that local tissue hypothyroidism may rather support tumoral transformation and even initiate enhanced cancerous proliferation. In contrast, studies on the effects of systemic hypothyroidism and cancer risk show opposite or conflicting results. For instance, hypothyroidism was associated with increased risk of hepatocellular carcinoma, and cancers of lung and prostate. In case of breast cancer, different studies reported that hypothyroidism was linked to reduced risk of the disease in humans while when studied in mice, hypothyroidism reduced tumour growth but enhanced metastasis. Finally, studies performed on cell line and mouse xenografts of thyroid and kidney cancers showed that inhibition of integrin-mediated thyroid hormone signaling can effectively result in inhibition of tumour growth.

All these studies show that the role of thyroid hormones in carcinogenic process is complex and presumably depends on type of cancer and extra- or intracellular effects of TH signaling. The results showing benefits from the use of TH pathway genes in cancer treatment and diagnosis suggest that further and more in depth studies are needed.

